# Maximum Entropy Probability Density Principle in Probabilistic Investigations of Dynamic Systems

**DOI:** 10.3390/e20100790

**Published:** 2018-10-15

**Authors:** Jiří Náprstek, Cyril Fischer

**Affiliations:** Institute of Theoretical and Applied Mechanics, Prosecká 809/76, 190 00 Prague 9, Czech Republic

**Keywords:** Boltzmann solution, Fokker–Planck equation, Gibbs entropy functional, maximum entropy probability density principle, random earthquake process, stochastically proportional system, 37H10, 60H10, 60H30, 60H35, 60K40

## Abstract

In this study, we consider a method for investigating the stochastic response of a nonlinear dynamical system affected by a random seismic process. We present the solution of the probability density of a single/multiple-degree of freedom (SDOF/MDOF) system with several statically stable equilibrium states and with possible jumps of the snap-through type. The system is a Hamiltonian system with weak damping excited by a system of non-stationary Gaussian white noise. The solution based on the Gibbs principle of the maximum entropy of probability could potentially be implemented in various branches of engineering. The search for the extreme of the Gibbs entropy functional is formulated as a constrained optimization problem. The secondary constraints follow from the Fokker–Planck equation (FPE) for the system considered or from the system of ordinary differential equations for the stochastic moments of the response derived from the relevant FPE. In terms of the application type, this strategy is most suitable for SDOF/MDOF systems containing polynomial type nonlinearities. Thus, the solution links up with the customary formulation of the finite elements discretization for strongly nonlinear continuous systems.

## 1. Introduction

A seismic wave that propagates horizontally in the upper subsoil layers mostly has a stochastic character, where its predominance increases with the distance from the earthquake epicenter. This fact originates from the filtering of a seismic wave through a continuum with randomly distributed non-homogeneity. A large number of records and data analyses have confirmed this approach. In principle, the seismic wave has three-dimensional characteristics composed of a number of partial waves that propagate with different velocities and other attributes, which change with the distance from the epicenter.

The propagation of a seismic wave through a non-homogeneous continuum and its transformation into a nearly stochastically homogeneous process has been investigated widely in theoretical and experimental studies. These have been conducted under laboratory conditions using special equipment, or directly in situ under the conditions of a real earthquake field. Many previous studies have addressed this issue in recent decades, including seismological investigations (e.g., [1,2]), numerical analyses (e.g., [3]), and theoretical analyses with overlap from seismic to poly-crystalline material features (e.g., [4,5]) or waves generated due to scattering on randomly rough inter-layer limits (e.g., [6]).

If the structure is large in a plane view and its horizontal dimensions are comparable or higher than the typical seismic wavelengths, then the distortion due to excitation processes acting on individual points of the basement should be considered (see the studies cited above). In particular conditions, either the phase shift of the excitation process in different points of the basement needs to be considered, or different random processes with non-zero cross-correlation are assumed to act on the corresponding points (e.g., see [7,8], where many additional attributes are also discussed).

In general, seismic processes are non-stationary. This fact is well documented and considered in models of the dynamic behavior of structures subjected to this special type of excitation (see [9,10] and monographs devoted to stochastic differential equations (SDEs) and random processes with engineering applications, such as [11,12]). Seismic processes are non-stationary in terms of both their amplitude and frequency, but the rough upper estimate of a structural response can be evaluated by considering a hypothetical stationary random process with suitable parameters [7,8]. Indeed, regardless of their non-stationarity, seismic processes generally have special characteristics, which is clear from the usual shape of their spectral density as a function of the time and frequency. Moreover, it is important to remember the great efforts that have been made to understand the basic mechanisms of these processes and the possibility of simplifying them as stationary events, such as the specific tools employed for their investigation [13,14], empirical mode decomposition analysis [15], and suitable methods for the analysis of seismic signals and data mining (e.g., [16,17]).

**Figure 1:**
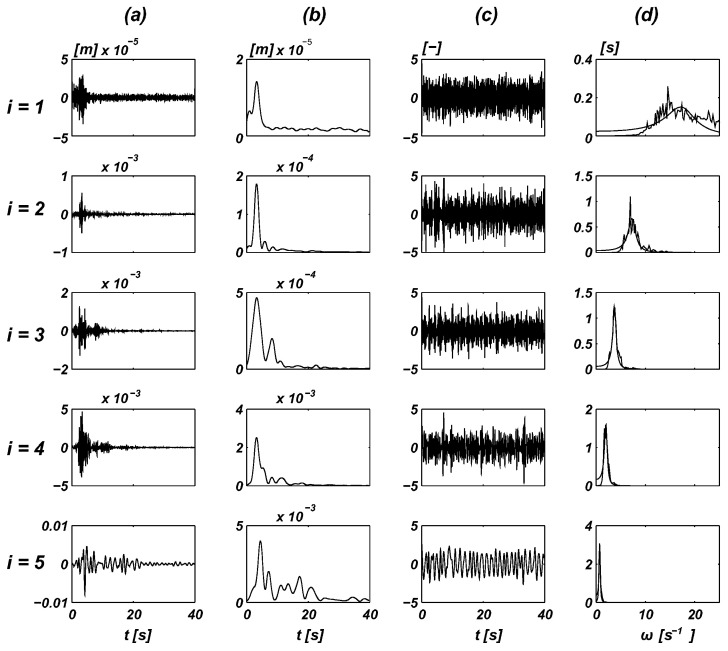
Non-stationary stochastic characteristics of an original record of a seismic shock—decomposition of the Sierra Madre earthquake record (five components *i* = 1–5). (**a**) Narrow band components $m_{i}\left(t\right)\cdot v_{si}\left(t\right)$ [m]. (**b**) Amplitude modulation $m_{i}\left(t\right)$ [m]. (**c**) Stationary process $v_{si}\left(t\right)$ [.]. (**d**) Spectral density $\Phi_{i}\left(\omega \right)$ [s] and its approximation (smooth curves).

**Figure 2:**
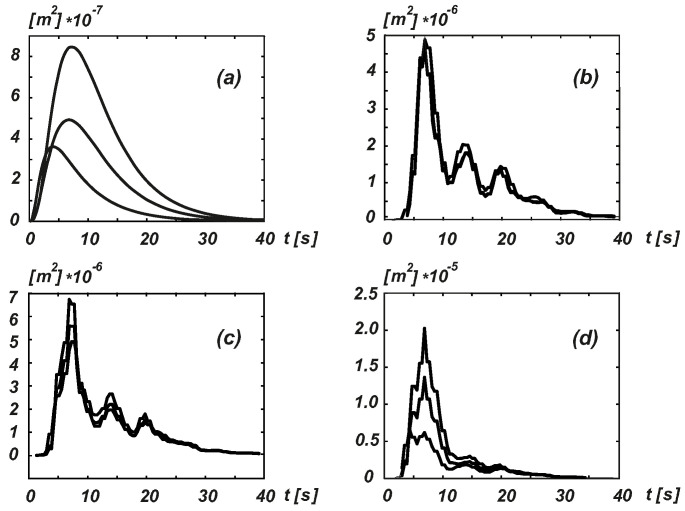
Displacement variance $\left[m^{2}\right]$ in the middle node of a simplified bridge (lumped mass, five degrees of freedom) subjected to excitation in the Sierra Madre earthquake record. (**a**) Simple amplitude modulation—exponential. (**b**) Simple amplitude modulation—$B^{N}$ spline. (**c**) Split Fourier spectra—$B^{N}$ spline. (**d**) Wavelet decomposition—$B^{N}$ spline.

To demonstrate the characteristics of these processes and to justify this simplifying hypothesis, Figure 1 and Figure 2 show the displacement records plotted for the Sierra Madre earthquake taken at Altadena, Eaton Canyon Park station on 28 June 1991, with an epicentral distance of 49.3 km for the E–W component (for additional details of the analysis of this record, please refer to [18,19]). The assumption stated above is generally the standard in this area when the dynamic characteristics of seismic processes are considered. However, most recommendations are satisfied by only a certain static equivalent (e.g., see Eurocodes 8 and 9, and [20], as well as related studies). Another study [19] also indicated the discrepancies between the analytical results obtained based on rigorous stochastic dynamics and two standards.

The transmission of vibrations in the contact subsoil structure is usually characterized by significant nonlinearities in terms of both the stiffness and damping. Moreover, various types of nonlinearities emerge from vibration at large amplitudes when non-elastic and hysteretic processes are considered. Geometric nonlinearities cannot be neglected in some configurations (e.g., see [21,22]). The analysis of these nonlinearities is outside the scope of this study, but it is important to be aware that although the stationary part of the random excitation process can be considered Gaussian (e.g., see [1,9,10]). However, the response differs from a Gaussian probability density function (PDF) and it should be investigated in an appropriate manner (e.g., [11]).

By summing up the considerations given above, and assuming that the structure itself can be modeled as a multi-degree of freedom (MDOF) system, the following system of SDEs can be written:

(1) $$\frac{dx_{j}\left(t\right)}{dt}=f_{j}\left(x,t\right)+g_{jr}\left(x,t\right)w_{r}\left(t\right),\;j=1,\ldots ,2n,\;n—\mathrm{dynamic}\;\mathrm{degrees}\;\mathrm{of}\;\mathrm{freedom}$$

- $x={\left[x_{1},x_{2},‥,x_{2n}\right]}^{T}$—response components (space variables in the following): (i) $x_{2j-1}$—displacements; (ii) $x_{2j}$—velocities (*j* = 1, …, *n*),
- $w_{r}\left(t\right)$—Gaussian white noise with constant cross-density in terms of the stochastic moments $K_{rs}=E\left\{w_{r}\cdot w_{s}\right\};$r,s=1,m, m—number of acting noises
- E{·}—mathematical mean value operator in the Gaussian meaning,
- $f_{j}\left(x,t\right),g_{jr}\left(x,t\right)$—continuous deterministic functions of state variables x and time *t*; j=1,2n.

The SDE system in Equation (1) can be considered a governing differential system, which allows us to model the various responses of an engineering structure for many types of seismic random processes. Various modifications of the system in Equation (1) can be found in numerous monographs that deal with deterministic or stochastic versions of this basic mathematical model. Rigorous mathematical analyses of its basic properties, limitations of its applicability, and the assumptions that should be satisfied in order for the system to be meaningful have been presented in previous studies (e.g., see [11,12,23,24]). It should be noted that $w_{r}\left(t\right)$ must be Gaussian processes or they need to be prepared using a suitable filtering method, or by considering the specific spectral properties of the processes considered (e.g., see [1,2,23]).

If Equation (1) is linear and the excitation processes are only of additive type, then various modifications of the spectral and correlation type solution methods are applicable because the response of a linear system under additive Gaussian excitation is a vector of Gaussian processes. Therefore, only the first two stochastic moments (mean value vector and variance square matrix) need to be determined to obtain a full description of the stochastic system response considered. Other dynamic systems produce non-Gaussian responses and they should be investigated using different methods.

The Fokker–Planck Equation (FPE) appears to be the most frequently used tool for solving the cross-PDF of a dynamic system excited by a vector of random processes $w_{r}\left(t\right)$, as shown in Equation (1).

If the input processes $w_{j},w_{r}$ can be considered Gaussian, then the respective FPE for an unknown PDF with the variables x,t can be associated with Equation (1) as follows:

(2) $$\frac{\partial p(x,t)}{\partial t}=-\frac{\partial }{\partial x_{j}}\left(\kappa_{j}\left(x,t\right)\cdot p\left(x,t\right)\right)+\frac{1}{2}\frac{\partial^{2}}{\partial x_{j}\partial x_{k}}\left(\kappa_{jk}\left(x,t\right)\cdot p\left(x,t\right)\right)$$

(3) $$\kappa_{j}\left(x,t\right)=\lim_{\Delta t\to 0}E\left\{\Delta x_{j}\right\}/\Delta t;\;\kappa_{jk}\left(x,t\right)=\lim_{\Delta t\to 0}E\left\{\Delta x_{j}\cdot \Delta x_{k}\right\}/\Delta t$$

- $\kappa_{j}\left(x,t\right)$—drift coefficients;
- $\kappa_{jk}\left(x,t\right)$—diffusion coefficients, j,k=1,…,n.

Random inputs affect the system as additive or multiplicative processes. Many previous studies have considered this well-known partial differential equation, which has mostly linear characteristics, although more complex definitions also exist (for a comprehensive explanation, see [11,12,23,24,25]). The processes $w_{r}\left(t\right)$ can be considered stationary or non-stationary. In principle, they do not need to be perfectly delta-correlated but quite complicated formulae are required for their evaluation (e.g., see [11,23]), and they affect the system as additive or multiplicative processes.

Analytical and semi-analytical methods for obtaining the FPE solution have been described previously. One of the most important was given by [26]. Several studies specifically addressed the analytical or semi-analytical FPE solution procedures (e.g., [27,28]). We note that although there are boundaries between the relevant groups, some of them are rather blurred. Fourier decomposition-based procedures are used particularly widely in theoretical physics, where they are based on a separability p(x,t)=p(x)·φ(t), and the drift and diffusion coefficients should be time independent. A Boltzmann type solution is used frequently as a basic step in the subsequent analysis by employing perturbation techniques. The Galerkin–Petrov method is probably the most general and suitable approach for investigating the majority of problems formulated in terms of the FPE. Many other specific case-oriented methods are based on the idea of completing various potentials, first integrals and their combinations, free parameter fitting, and other operations. Despite the strengths of these methods and the excellent results obtained, they have many disadvantages due to the highly limited dimensionality of FPE, the possibility of configuring the boundary conditions, and problems when analyzing any non-stationary problems.

Numerical methods are a suitable alternative and various generally formulated procedures with respect to large dimensionality have been presented and tested (e.g., see [29]). Nevertheless, the finite element method (FEM) seems to be the most promising approach (e.g., see [30,31,32] and the references therein). The FEM is affected by several common shortcomings in the same manner as every numerical method but it provides options that are lacking in other methods such as the possibility of analyzing transitions and quasi-periodic processes.

Among the strategies mentioned, one principle has been neglected in recent years, possibly due to the overwhelming dominance of purely numerical methods. This principle is based on the Gibbs entropy of probability and it lies between analytical and numerical methods. Indeed, it can serve as the backbone of a large group of FPE solution methods. We provide some examples of this method in the following.

## 2. Gibbs Entropy of Probability

Entropy is a physical idea that was introduced into the theory of thermodynamic systems in the 19th century as a quantity for characterizing some macroscopic property, and it is indispensable for describing the relationships among mechanical, thermic, and other fields. The statistical concept of micro-mechanics absorbed this concept very early, where Gibbs and other theoreticians demonstrated how the entropy with a suitable meaning could be used for statistically describing the uncertainties of internal processes. Subsequent studies by Jaynes [33] and Kullback [34] integrated information theory with statistical mechanics (on micro-level) to allow the possibility of expressing an indeterminacy based on the entropy. In principle, they showed that the actual PDF of a system state supplies the maximum with respect to a certain functional among all other PDF distributions belonging to the admissible states. Based on such a procedure the so-called *b*-value in the Gutenberg–Richter law for earthquakes, which states the (cumulative) number of earthquakes with magnitude greater than *M* occurring in a specified area and time is given by $\left(N\left(>M\right)\approx 10^{-bM}\right)$, was determined in [35] by applying the maximum entropy principle to data analyzed in a new time domain termed natural time [36]. Thus, the response PDF can be found for a particular system provided we have a complete description of the system with the boundary and initial conditions.

This principle led to a revolution in quantum mechanics and thermo-mechanics. The macroscopic properties of a particular system can be described further in terms of statistical parameters based on the properties and state of its microscopic particles. This advance represented a significant step change in solid-state physics and many other areas. Information theory is very closely related to the random characteristics of micro-particles and advanced rapidly as a consequence. This principle has been applied in many disciplines such as data mining and signal processing (as well as stochastic resonance and other areas; e.g., see [37,38,39]), sea level description [40] (indirect information), and laser technology (e.g., [41]). The maximum entropy principle has proved very useful in studies of earthquake recurrence (e.g., see [42]) as well as physiology and applications in human medicine (e.g., [43]).

The consideration of entropy (as the entropy of probability) in investigations of the dynamics of macroscopic systems in engineering occurred in the late 1980s and early 1990s (e.g., see [44,45,46,47,48,49]). The delay in the inclusion of entropy compared with solid-state physics and other disciplines may have been due to the somewhat opposite formulation of the problem (from known elements to the unknown PDF of the system response), and thus the use of different mathematical formulation strategies and the subsequent solutions. Projecting entropy into the mathematical formulation means that the unknown PDF is searched for under certain boundary conditions and auxiliary constraints are associated via an unknown Lagrange multiplier. The specific solution procedures appear to be a compromise between (semi-) analytical and numerical methods.

Let us formulate a closed mathematical system that allows us to receive satisfactory information about the PDF of the system response given by Equation (1) together with adequate boundary and initial conditions. The basic principle is associated with the functional introduced by Gibbs [50] in the form intended for the domain of micro-particles, as follows.

(4) $$S=-\kappa_{B}\int_{\left(x\right)}p\left(x,t\right)\cdot \mathrm{lg}\;p\left(x,t\right)\cdot dx,$$

- $\kappa_{B}$—Boltzmann constant assigning *S* its physical meaning,
- $x=|x_{1},x_{2},\cdots ,x_{2n}{|}^{T}$, see Equation (1)

The entropy of the state of the system in the form of Equation (4) was introduced based on a theoretical study of the probability of the state of the gas particle distribution in a closed vessel (see [51]). Except for the multiplication constant, the same expression can be obtained if we examine the rate of indeterminateness in the description of an object or phenomenon in information theory [33].

The quantity in Equation (4) is a measure of the indeterminateness at moment *t* and it characterizes the incompleteness of our information regarding the processes occurring at the microscopic level.

In the case of irreversible processes, the entropy of the state of disequilibrium increases continuously with time. The state of equilibrium for a perfectly isolated system is characterized by the maximum entropy because it holds that:

(5) $$\frac{\partial S}{\partial t}\geq 0.$$

Consequently, the transition to the state of equilibrium is characterized by an increase in indeterminateness and a decrease in the information level. Thus, in terms of Equation (5), the state of equilibrium has an extreme character, as follows.

(6) $$S_{stac.}=max$$

Therefore, the probability density of the state for the equilibrium does not depend on time. The entropy value is maximized compared with all preceding moments for the transition state directed at attaining this equilibrium state. However, the equilibrium state does not need to be unique in the final volume of the phase space because it corresponds to nonlinear systems.

Similar to all other methods, this procedure is not fully universal and it may be recommended as effective for only a special group of systems. This is due to the basic philosophy of the principle and mainly because of the need to obtain a solution with sufficient effort to provide transparent results that allow a physical interpretation. However, some potential problems affect the appropriate techniques used for searching for an extreme of the Gibbs functional. These approaches can work correctly in the case of systems with highly distinct nonlinear normal modes (NNMs) with minimal mutual interaction and the marginal influence of local modes. The problems are closely related to the possibility of effectively employing Boltzmann type solutions (e.g., see [23]) as a starting approximation for subsequent iterative steps.

The practical application of the functional based on Equation (4) to problems in statistical physics is based on the fact that the probability density p(v,t) is considered to be a known quantity determined in experiments, or based on independent partial considerations according to the specific conditions of the system. For instance, Gibbs introduced the so-called micro-canonic probability distribution for the adiabatic process in gases. However, the statistical mechanics of systems must be based on the assumption that the probability density of a response is an unknown function, particularly for nonlinear systems.

If the input or output are not characterized by the presence of white noise, Equation (6) is not sufficient for determining p(x,t), and it does not contain any information about the structure of the system. However, every potential system is described by the equations of motion Equation (1), with which p(x,t) must also comply. Therefore, we can formulate the following variation of the problem as finding the cross-probability density p(x) of a stationary response process where the functional Equation (4):

(7) $$S=-\int_{\left(x\right)}p\left(x\right)\cdot \mathrm{lg}\;p\left(x\right)dx,$$

attains the maximum and the equations of motion Equation (1) comply with the statistical meaning, such as the meaning of the stochastic moments. Thus, we seek the weak solution of the problem in terms of the stochastic meaning, which differs from a weak solution in terms of the classical meaning. In Equation (7), we can use $\kappa_{B}=1$ because the entropy will be evaluated only as a relative value when searching for the maximum from a set of admissible functions p(x) and not as an absolute physical quantity. Some attributes considered in extreme search by [52] can provide inspiration, but care should be taken because the functional Equation (7) is not quadratic. Therefore, basic theorems regarding the extreme existence do not hold.

## 3. Formulation of the Secondary Constraints

As mentioned in Section 2, following Equation (6), the maximum of *S* should be searched for with respect to the secondary constraints. The secondary constraints on the extreme may be selected in various ways, but they should specify an exact structure for the dynamic system considered in every case. This structure is primarily given by Equation (1). In terms of the stochastic meaning, the most natural system character specification follows from the FPE, i.e., Equation (2) with a scalar unknown quantity PDF. The coefficients of the FPE include all of the characteristics of Equation (1).

For the stationary case of the FPE, Equation (2) has the form:

(8) $$\frac{\partial \left(\kappa_{j}\left(x\right)p\left(x\right)\right)}{\partial x_{j}}-\frac{1}{2}\frac{\partial^{2}\left(\kappa_{jk}\left(x\right)\cdot p\left(x\right)\right)}{\partial x_{j}\partial x_{k}}=0,$$

where $\kappa_{j}\left(x\right),\kappa_{jk}\left(x\right)$, j,k=1,…,n—drift and diffusion coefficients (moments of the 1st and 2nd orders) of the *n*-dimensional processes, which are now limited to Gaussian white noise independent of time *t*.

In this case, the drift and diffusion coefficients in Equation (3) can be simplified significantly and written as closed formulae:

(9) $$\kappa_{j}\left(x\right)=f_{j}\left(x\right)+\frac{1}{2}K_{rs}\cdot g_{ls}\left(x\right)\frac{\partial_{jr}\left(x,t\right)}{\partial x_{l}}\;;\;\kappa_{jk}\left(x\right)=K_{rs}\cdot g_{jr}\left(x,t\right)g_{ks}\left(x,t\right),$$

where:

- $K_{rs}=E\left\{w_{r}\left(t\right)w_{s}\left(t\right)\right\}$—matrix of white noise intensities (independent of time).
- E—operator of mathematical mean value.

It should be noted with respect to Equation (3) that the drift and diffusion coefficients can be evaluated also in more complicated cases when the spectral densities of input processes are not constant. The following formulae can be used:

(10) $$\begin{array}{cc} \kappa_{j}\left(x\right) & =f_{j}\left(x\right)+\int_{-\infty }^{0}g_{ls}\left(x_{t+\tau },t+\tau \right)\frac{\partial }{\partial x_{l}}g_{jr}\left(x_{t},t\right)R_{rs}\left(\tau \right)d\tau ,\\ \kappa_{jk}\left(x\right) & =\int_{-\infty }^{\infty }g_{jr}\left(x_{t},t\right)g_{ks}\left(x_{t+\tau },t+\tau \right)R_{rs}\left(\tau \right)d\tau . \end{array}$$

where $R_{rs}\left(\tau \right)$ is a general cross-correlation function of processes $w_{r},w_{s}$. When the correlation function degenerate to Dirac functions $R_{rs}=K_{rs}\delta \left(\tau \right)$ as it corresponds with white noises, expressions Equation (10) turn into (9). Application of formulae Equation (10) for processes with bi-modal spectral densities is shown for instance in [11] or [53]. However, from the viewpoint of demonstration of the maximum entropy principle in dynamics this aspect does not play a significant role and the reduced relations (9) are valid.

In the case considered, the probability density must satisfy the boundary conditions as follows:

(11) $${\left.p(x)\right|}_{x_{j}\to \pm \infty }=0;\;{\left.\frac{\partial p(x)}{\partial x_{j}}\right|}_{x_{j}\to \pm \infty }=0$$

If we substitute according to Equation (1) into coefficients $\kappa_{j},\kappa_{jk}$ following Equation (9), or into Equation (3) with the time omitted, we obtain the following.

(12) $$\begin{array}{cc} \kappa_{j}= & -f_{j}\left(x_{1},\cdots ,x_{n}\right)\\ \kappa_{jk}= & g_{jl}\left(x_{1},\cdots ,x_{n}\right)g_{lk}\left(x_{1},\cdots ,x_{n}\right)s_{ll} \end{array}$$

In these conditions, we can consider Equation (8) as the secondary constraints on the functional Equation (7) extreme. Thus, the problem has been determined and it is possible to begin searching for the stationary point or points, if any exist.

In this particular case, it is best to formulate the secondary constraints in the form of stochastic moments for clarity. The equations can be deduced from the FPE. In the case of a linear system excited by a Gaussian process, if the solution exists, then using only the first and the second moments will be sufficient for its full description. In the case of nonlinear systems, an infinitely large system of mutually bound equations will emerge and this type of system is generally nonlinear. In general, the series of stochastic moments must be convergent and the cumulants should then be considered instead. Nevertheless, for brevity, let us assume that the stochastic moments are convergent, which allows us to restrict the system using some criterion (e.g., declaring the moments only up to a certain order as independent and the moment of a higher order as their functions, e.g., the Gaussian closure in the simplest case).

Thus, if the number of moments and the number of equations is limited, it is generally possible to find a high number of probability densities p(x) that satisfy these equations. If the number of moments remaining in the problem increases, then the size of the set from which we seek the maximum also decreases. In the limit, this set will contain only one element in an arbitrarily small neighborhood and the functional Equation (7) will only characterize the given state. However, the whole phase space may contain a countable number of such stationary points, which correspond to different equilibrium states according to the different levels of the functional value in Equation (7). In this case, the whole philosophy of the variational solution to p(x) is similar to the philosophy of the direct variational methods used in the theory of elasticity as an example. It should be noted that this overall approach is only applicable close to the stationary point where the state of the system can be considered at least approximately quasi-stationary, and thus time can be considered as a parameter. In addition, it should be noted that the notion of entropy can be extended by expanding the manipulation space ensuring non-stationary processes can also be investigated (e.g., see [54]). However, only stationary problems are considered in the present study and this extension is not addressed.

## 4. One-Component Systems

### 4.1. Directly Finding the Extreme—Boltzmann’s Solution

The one-dimensional diffusion process is described by a stochastic equation of first order:

(13) $$\dot{x}+f\left(x\right)=w\left(t\right),$$

where f(x) is a smooth integrable function. The system is excited by Gaussian white noise w(t) with intensity *s* on the right side. This equation (reduced Langevin type) appears frequently in applied physics and engineering, particularly in earthquake-related problems regarding wave propagation in a significantly pronounced hysteretic continuum where inertia forces are marginal and dissipation forces are dominant.

The coefficients Equation (12) associated with Equation (13) have the form:

(14) $$\kappa_{1}\left(x\right)=f\left(x\right),\;\kappa_{11}=s^{2},$$

therefore, the FPE reads:

(15) $$\frac{\partial }{\partial x}\left(f(x)p(x)\right)+\frac{1}{2}s\frac{\partial^{2}p\left(x\right)}{\partial x^{2}}=0.$$

Equation (15) with the respective boundary conditions unequivocally satisfies the Boltzmann type solution:

(16) $$p\left(x\right)=C\exp\left(-\frac{2}{s}\int f\left(x\right)dx\right).$$

In this process, it is necessary to impose certain conditions on f(x) because p(x) must comply with the requirements imposed on the density of the probability distribution (normalization, existence of moments, etc.).

However, Equation (16) can also be obtained by searching for the maximum of the functional Equation (7) with the secondary constraint Equation (15), which can be satisfied by the weak solution in terms of the statistical meaning. For odd functions f(v), it is possible to obtain a derivation from Equation (15) for statistical moments:

(17) $$\int_{-\infty }^{\infty }\left(x^{k}f\left(x\right)-\frac{1}{2}skx^{k-1}\right)p\left(x\right)dx=0.$$

In addition, it is necessary to respect the normalization constraint:

(18) $$\int_{-\infty }^{\infty }p\left(x\right)dx=1.$$

Thus, the maximum of the functional Equation (7) is limited by the secondary constraints Equations (17) and (18). Using the Lagrange multipliers, we arrive at the problem of the unconditional extreme of the functional:

(19) $$S=-\int_{-\infty }^{\infty }p\left(x\right)\mathrm{lg}p\left(x\right)dx-\left(\lambda_{0}-1\right)\int_{-\infty }^{\infty }p\left(x\right)dx-\int_{-\infty }^{\infty }p\left(x\right)\sum_{1}^{\infty }\lambda_{k}\left(x^{k}f\left(x\right)-\frac{1}{2}skx^{k-1}\right)dx,$$

where $\lambda_{0}$, $\lambda_{k}$ are the unknown Lagrange multipliers. $\lambda_{0}$ is related to the normalization of the PDF.

The annulment of the variation for expression Equation (19) with respect to p(x) using the continuity of the integrand in Equation (19) yields the general expression for the extreme, as follows.

(20) $$p\left(x\right)=e^{-\lambda_{0}}\cdot \exp\left(-\sum_{k=1}^{\infty }\lambda_{k}\left(x^{k}f\left(x\right)-\frac{1}{2}skx^{k-1}\right)\right)$$

The coefficient $\lambda_{0}$ can be determined from the normalization constraint and $\lambda_{k}$ by re-substituting Equation (20) into Equation (17). In this manner, we arrive at the following relation after several modifications.

(21) $$\sum_{k=1}^{\infty }\lambda_{k}\left(x^{k}f\left(x\right)-\frac{1}{2}skx^{k-1}\right)=\frac{2}{s}\left(\frac{1}{1!\cdot 2}f_{0}^{\prime }x^{2}+\frac{1}{3!\cdot 4}f_{0}^{\prime \prime \prime }x^{4}+\frac{1}{5!\cdot 6}f_{0}^{v}v^{6}+\cdots \right)$$

However, the right-hand side of Equation (21) is the Taylor’s series of the indefinite integral for the odd continuous function f(x). Thus, Equation (19) has a form that is entirely identical to Equation (15), which we aimed to prove. Therefore, according to Equation (16), p(x) determines the extreme value of the functional Equation (7) while complying with the secondary constraint Equation (15). In this simple case, we have successfully complied with an infinite number of these conditions (in terms of the moments). Therefore, the selection of the set is reduced to a single element Equation (16), which simultaneously satisfies the equation or the secondary constraint Equation (15) in terms of the statistical meaning as well as the classical solution.

The secondary constraints mentioned several times in this study are symbolically treated in the form of the stationary version of the FPE in Equation (8). This method is preferable given that the basic considerations have general characteristics. The decomposition of the stochastic moments is preferable for obtaining practical solutions to particular problems (although other approaches are also suitable). We consider several applications of this strategy in the following sections of this study. Therefore, it is useful to consider the basic character of convergence when manipulating using this decomposition.

Let us return to the series comprising Equation (17) and the normalization constraint Equation (18). We assume the cubic characteristic $f\left(x\right)=ax+bx^{3}$. The first approximation is obtained for k=1, which means that:

(22) $$p\left(x\right)=C\exp\left(-\lambda_{1}\left(x\left(ax+bx^{3}\right)-\frac{1}{2}s\right)\right).$$

By introducing Equation (22) into Equation (17) for k=1, we obtain an algebraic equation for the multiplier $\lambda_{1}$:

(23) $$\int_{-\infty }^{\infty }\left(x\left(ax+bx^{3}\right)-\frac{1}{2}s\right)C\exp\left(-\lambda_{1}\left(x\left(ax+bx^{3}\right)-\frac{1}{2}s\right)\right)dx=0.$$

**Figure 3:**
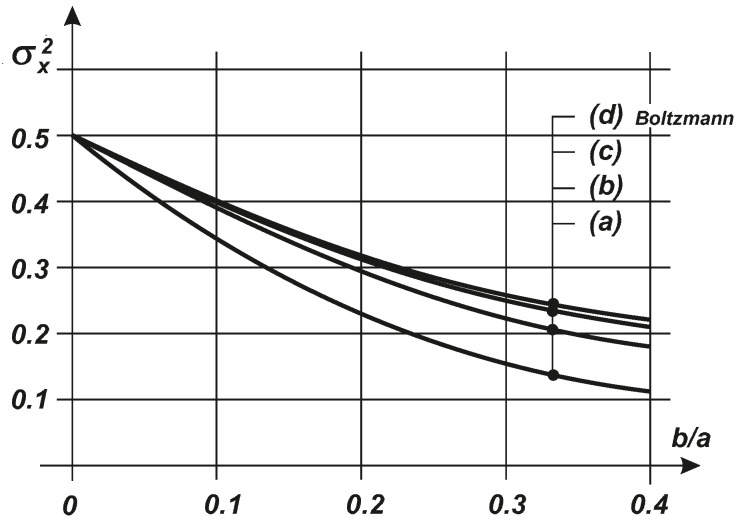
Comparison of the variance $\sigma_{x}^{2}$ evaluated for: (**a**) system with stochastic linearization; (**b**) system with the maximum entropy of probability, as the first approximation q=1; (**c**) the second approximation q=2; (**d**) Boltzmann solution.

The integrals in Equation (23) can be evaluated exactly in cylindric functions, e.g.,

(24) $$\int_{-\infty }^{\infty }\exp\left(-\lambda_{1}\left(ax^{2}+bx^{4}-\frac{1}{2}s\right)\right)dx=\sqrt{\frac{a}{4b}}\exp\left(\frac{1}{2}\lambda_{1}\left(s+\frac{a^{2}}{4b}\right)\right)\cdot K_{1/4}\left(\frac{\lambda_{1}a^{2}}{8b}\right),$$

where $K_{1/4}\left(\frac{\lambda_{1}a^{2}}{8b}\right)$ is the Macdonald function. Clearly, in the case of common applications, the integrals in Equation (24) should be evaluated numerically, which causes some difficulties because unknown values or the values of $\lambda_{k}$ are parts of the arguments of the exponential functions. Nevertheless, for this example, we can plot (see Figure 3) curves where we illustrate the variance $\sigma_{x}^{2}$ of the response *x* for the stochastically linearized case in curve (a), those evaluated at the levels of the first and second approximations q=1,2 in curves (b) and (c), respectively, and those obtained with the exact Boltzmann solution using Equation (16) in curve (d). Even the very simple approximation obtained using the probability entropy maximum strategy is effective and it converges very rapidly to the exact solution. The results obtained for the second approximation and the Boltzmann solution are almost indistinguishable.

### 4.2. First Order System with Complex Characteristics

We illustrate the effectiveness of the maximum entropy probability principle for another case with a one-component response process, which is produced by a nonlinear system of first order with relatively complex characteristics:

(25) $$\frac{dx}{dt}+ax+bx^{2}+cx^{3}\mathrm{lg}\frac{\left|x\right|}{\alpha }=\;w\left(t\right),$$

where

- a,b,c>0,α>0 are real coefficients,
- w(t) is a Gaussian white noise with intensity *K*.

The detailed analysis was reported by Sobczyk and Trebicki [46,47]. A small number of previous studies focused on the principle result obtained with respect to Equation (25). In addition to these studies, mathematical models related to Equation (25) can be found in seismicity research in investigations of the passage of nonlinear waves through a well-saturated medium containing a liquid material. In addition, the aero-elasticity of systems with super-critical damping is related to an equation of this type, as well as the theory of diffusive chemical processes.

The relevant drift and diffusion coefficients are:

(26) $$\kappa_{1}=-\left(ax+bx^{2}+cx^{3}\mathrm{lg}\frac{\left|x\right|}{\alpha }\right),\;\kappa_{11}=s^{2}.$$

**Figure 4:**
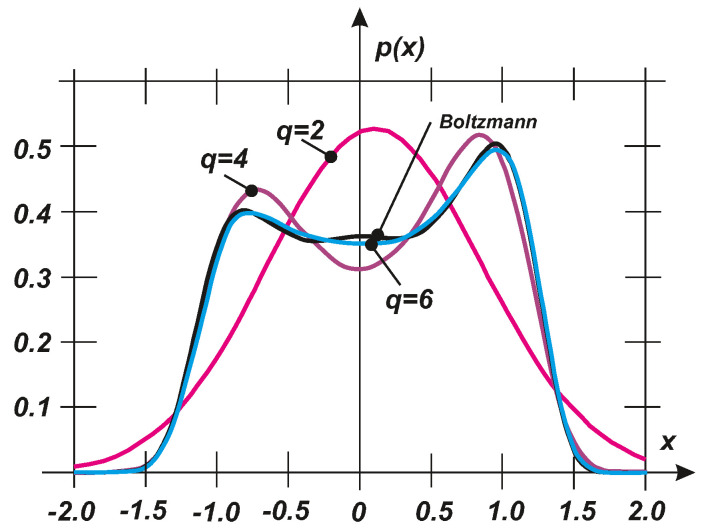
Comparison of the exact PDF (Boltzmann) and the PDFs determined using the given stochastic moments (q=2,4,6).

With respect to the Boltzmann general solution for Equation (16), it holds that:

(27) $$p\left(x_{1}\right)=C\exp\left(-\frac{x^{2}}{s^{2}}\left(a+\frac{2}{3}bx+\frac{1}{2}cx^{2}\left(\mathrm{lg}\frac{\left|x\right|}{\alpha }-\frac{1}{4}\right)\right)\right),$$

where *C* is a normalizing constant. Using Equation (27), the exact stochastic moments can be evaluated to demonstrate the fidelity of an exact PDF characterization, as plotted in Figure 4 for the exact PDF, where Equation (27) and the PDFs are determined based on q=2,4,6 first stochastic moments. The specific values of the parameters are as follows: a=0.5, b=−0.25, c=5.2, α=1.0, s=1.0. For q=2, a Gaussian distribution is provided and thus the result is unacceptable. Nevertheless q=4 and q=6 exhibit very rapid convergence and they can represent a satisfactory approximation of the PDF according to Equation (27). It should be noted that the exact PDF exhibits approximately bi-modal characteristics. Therefore, the basic approximation for q=2 should pass very quickly from the uni- to bi-modal type of the PDF.

**Figure 5:**
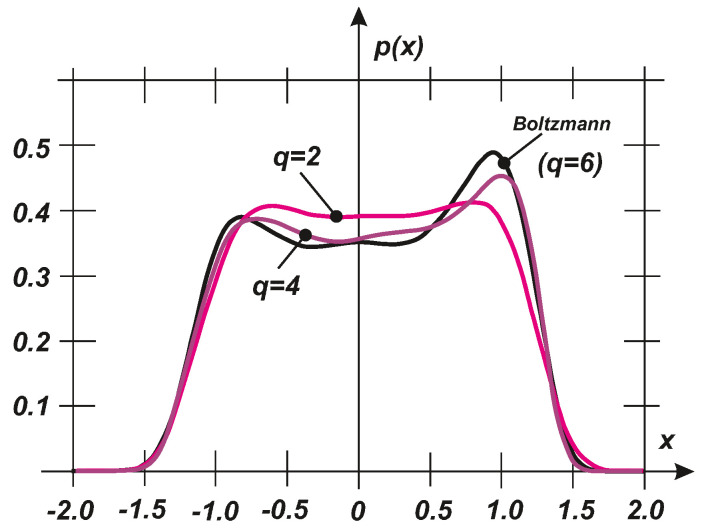
Comparison of the exact PDF (Boltzmann) and the PDFs determined with various numbers of stochastic moment equations (q=2,4,6).

Next, we consider a limited number of equations for the first stochastic moments (q=2,4,6). According to the general method for deriving the equations for the stochastic moments of the relevant Ito equations (e.g., [11,12,23]), the following hierarchy of equations for stochastic moments can be deduced:

(28) $$\int_{-\infty }^{\infty }\kappa_{1}x^{k-1}dx+\frac{1}{2}\left(k-1\right)\int_{-\infty }^{\infty }\kappa_{11}^{1/2}x^{k-2}dx=0,\;k=1,2,\ldots ,q,$$

where $\kappa_{1},\kappa_{11}$ are given by Equation (26). The standard codes using a homotopy background were used to evaluate some example results. The values of the parameters were identical to those employed above: a=0.5, b=−0.25, c=5.2, α=1.0, s=1.0. The exact PDF and the PDFs corresponding to two and four given moments are shown in Figure 5. Clearly, the PDF determined using the maximum entropy principle is very close to the exact PDF even when using four stochastic moment equations, Equation (28). The convergence can be assessed using the objective Kullback–Leibler divergence criterion (see [34,55]):

(29) $$\Delta_{pp^{*}}=\int_{-\infty }^{\infty }p\left(x\right)\cdot \mathrm{lg}\frac{p(x)}{p^{*}\left(x\right)}dx$$

where

- p(x)—the exact PDF,
- $p^{*}\left(x\right)$—the approximate PDF corresponding to q=2,4,6, respectively.

The respective divergence parameter $\Delta_{pp^{*}}$ has values of:

(30) $$\Delta_{pp^{*}}^{\left(2\right)}=0.01053260,\;\Delta_{pp^{*}}^{\left(4\right)}=0.00014443,\;\Delta_{pp^{*}}^{\left(6\right)}=0.00000278,$$

which demonstrates the rapid convergence of the approximate PDFs to the exact solution of the Boltzmann type. There is no clear difference between the solutions based on the Boltzmann type method and q=6 maximum entropy (see Figure 5), as also shown in Section 4.1 and Figure 3.

## 5. Two-Component Systems

### 5.1. System with Diffusion Additive Excitation

**Figure 6:**
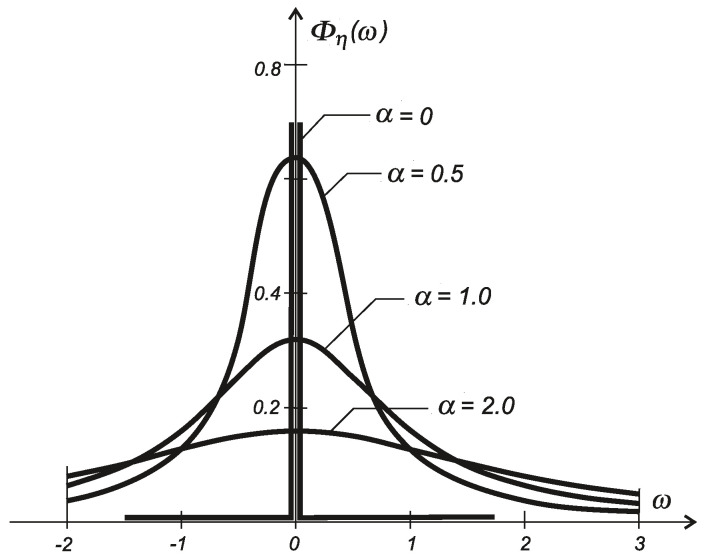
Spectral density $\Phi_{\eta }\left(\omega \right)$ of a diffusion process η(t) for various parameters α.

We next describe a direct procedure for finding the PDF using the maximum entropy probability principle. We consider Equation (13):

(31) $$\dot{x}+f\left(x\right)=\eta \left(t\right),$$

where f(x) is a smooth function that is integrable on a limited interval and the process η(t) follows from a filtering of white noise through a linear differential filter of the first order:

(32) $$\dot{\eta }+\alpha \eta =w\left(t\right)v,$$

where $s=2\alpha \sigma_{\eta }^{2}$ is the intensity of the white noise w(t). The relevant spectral density of the η process (see Figure 6) is given by the formula:

(33) $$\Phi_{\eta }\left(\omega \right)=\frac{\sigma^{2}}{\pi }\frac{\alpha }{\omega^{2}+\alpha^{2}}.$$

Hence, we can define a two-component random process in terms of the meaning of the system in Equation (1):

(34) $$\begin{array}{cc} {\dot{x}}_{1} & =-f\left(x_{1}\right)+x_{2}\\ {\dot{x}}_{2} & =-\alpha x_{2}+w\left(t\right). \end{array}$$

The cross-PDF $p(x_{1},x_{2})$ of a stationary state can be described with respect to Equation (8) and the coefficients in Equation (9) by the following FPE:

(35) $$\frac{1}{2}s\frac{\partial^{2}p}{\partial x_{2}^{2}}+\alpha \frac{\partial (x_{2}p)}{\partial x_{2}}+\frac{\partial }{\partial x_{1}}\left((f\left(x_{1}\right)-x_{2})p\right)=0.$$

The closed form solution of the FPE in Equation (35) cannot be obtained. Therefore, we try to apply probability entropy maximization to the functional S (see Equation (7)).

Let us multiply Equation (35) successively by the factors $x_{1}^{2},x_{1}x_{2},x_{2}^{2}$, and subsequently apply integration on the infinite two-dimensional phase space. After several integration by parts steps with respect to the homogeneous boundary conditions in infinity for both components $x_{1},x_{2}$, we obtain three equations for the stochastic moments, which should be completed by the fourth equation to represent the constraints on normalization:

(36) $$\begin{array}{c} \iint_{-\infty }^{\infty }\left(x_{1}f\left(x_{1}\right)-x_{1}x_{2}\right)p\left(x_{1},x_{2}\right)dx_{1}dx_{2}=0,\\ \iint_{-\infty }^{\infty }\left(x_{2}f\left(x_{1}\right)+\alpha x_{1}x_{2}-x_{2}^{2}\right)p\left(x_{1},x_{2}\right)dx_{1}dx_{2}=0,\\ \iint_{-\infty }^{\infty }\left(\frac{1}{2}K-\alpha x_{2}^{2}\right)p\left(x_{1},x_{2}\right)dx_{1}dx_{2}=0,\\ \iint_{-\infty }^{\infty }p\left(x_{1},x_{2}\right)dx_{1}dx_{2}=1. \end{array}$$

The extended functional $S^{*}$ including the secondary and normalization constraints with the corresponding Lagrange multipliers is:

(37) $$\begin{array}{cc} S^{*}=-\iint_{-\infty }^{\infty }p\mathrm{lg}pdx_{1}dx_{2}-\iint_{-\infty }^{\infty } & \left[\left(\lambda_{0}-1\right)+\lambda_{1}\left(x_{1}f\left(x_{1}\right)-x_{1}x_{2}\right)+\right.\\ & \left.+\lambda_{2}\left(x_{2}f\left(x_{1}\right)+\alpha x_{1}x_{2}-x_{2}^{2}\right)+\lambda_{3}\left(\frac{1}{2}s-\alpha x_{2}^{2}\right)\right]pdx_{1}dx_{2}. \end{array}$$

According to the principle of maximum entropy, the PDF with the maximum entropy for the functional in Equation (37) has the form:

(38) $$p\left(x_{1},x_{2}\right)=C\exp\left[-\lambda_{1}\left(x_{1}f\left(x_{1}\right)-x_{1}x_{2}\right)-\lambda_{2}\left(x_{2}f\left(x_{1}\right)+\alpha x_{1}x_{2}-x_{2}^{2}\right)-\lambda_{3}\left(\frac{1}{2}s-\alpha x_{2}^{2}\right)\right],$$

where $C=\exp(-\lambda_{0})$.

By substituting Equation (38) back into Equation (36), we obtain four algebraic equations for $\lambda_{0}-\lambda_{3}$, which need to be solved for a particular case of the function $f(x_{1})$. This rather complicated algebraic system can be solved effectively by employing the same packages that are commonly used for homotopy continuation methods.

A good initial approximation can serve as a function corresponding to the Gaussian PDF and linear characteristic $f\left(x_{1}\right)=cx_{1}$:

(39) $$p_{0}\left(x_{1},x_{2}\right)=C\exp\left(-\frac{1}{2(1-r^{2})}\left(\frac{x_{1}^{2}}{\sigma_{1}^{2}}-\frac{2rx_{1}x_{2}}{\sigma_{1}\sigma_{2}}+\frac{x_{2}^{2}}{\sigma_{2}^{2}}\right)\right),$$

where

- $\sigma_{1}^{2},\;\sigma_{2}^{2}$—variances of $x_{1},\;x_{2}$ for the adjoint linear system,
- *r*—correlation of these coordinates.

**Figure 7:**
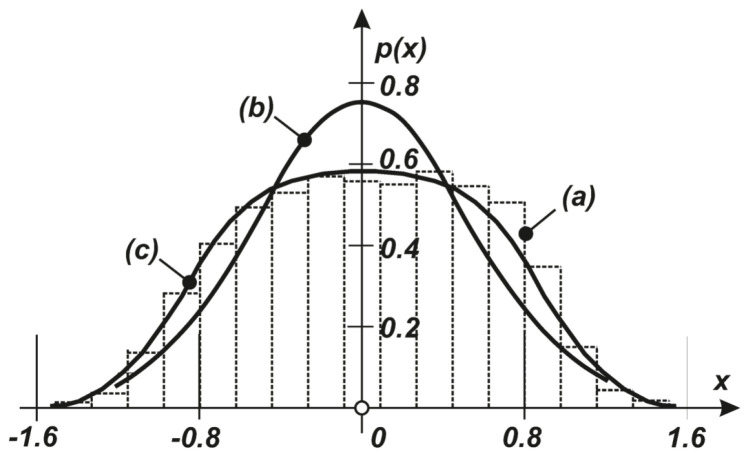
(a) PDF in $x_{1}$ following the histogram of the output process $x_{1}$. (b) Stochastic linearization. (c) Second moments approximation.

For the cubic nonlinearity of the system characteristic, $f\left(x_{1}\right)=ax_{1}+bx_{1}^{3}$ was evaluated fully as an example problem where the following values were used: $a=1,\;b=0.1,\;\alpha =0.5,\;\sigma_{1}^{2}=\sigma_{2}^{2}=s/2\alpha =1$. The plot in Figure 7 shows the one-dimensional PDFs reduced to coordinate $x_{1}$, as usual:

(40) $$p\left(x_{1}\right)=\int_{-\infty }^{\infty }p\left(x_{1},x_{2}\right)dx_{2}.$$

Figure 7 shows the PDF evaluated based on the stochastic linearization as curve (b), the approximation after maximization of the functional $S^{*}$ with Equation (37) as curve (c) at the level of q=2, and the raw histogram obtained for $x_{1}$ by stochastic simulation as the stepped curve in (a). A comparison of curves (a) and (c) clearly shows that the approximation of the second order moments provides acceptable results, whereas the linearization is far from correct.

### 5.2. Dynamic System with a Single Degree of Freedom

The vibrations of a system with one degree of freedom excited by white noise are described by a normal system of Equation (1) type, where n=1 and thus it contains two components $x_{1},\;x_{2}$:

(41) $$\begin{array}{c} {\dot{x}}_{1}=x_{2}\\ {\dot{x}}_{2}=\left(2\omega_{b}x_{2}+\omega_{0}^{2}x_{1}+f\left(x_{1}\right)\right)+w\left(t\right), \end{array}$$

where the argument *t* in $x_{1},\;x_{2}$ is omitted for brevity, therefore $x_{1}\left(t\right)=x_{1};\;{\dot{x}}_{1}\left(t\right)=x_{2}\left(t\right)=x_{2}$. Hence, the FPE is:

(42) $$-\frac{\partial (x_{2}p)}{\partial x_{1}}+\frac{\partial }{\partial x_{2}}\left(\left(2\omega_{b}x_{2}+\omega_{0}^{2}x_{1}+f\left(x_{1}\right)\right)p\right)+\frac{1}{2}s\frac{\partial^{2}p}{\partial x_{2}^{2}}=0,$$

where:

- $p=p(x_{1},\;x_{2})$—cross-probability density of $x_{1}\left(t\right)$, $x_{2}\left(t\right)$
- *s*—intensity of white noise w(t).

The solution may be obtained for Equation (42) with the boundary conditions in Equation (11) by using the Fourier method in the following form.

(43) $$p\left(x_{1},x_{2},t\right)=C\exp\left(-\frac{4\omega_{b}}{s}\frac{x_{2}^{2}}{2}\right)\cdot \exp\left(-\frac{4\omega_{b}}{s}\left(\frac{\omega_{0}^{2}x_{1}^{2}}{2}+\int f\left(x_{1}\right)dx_{1}\right)\right)$$

Thus, the displacement and displacement velocity are statistically independent. However, similarly to the preceding case, Equation (11) can be arrived at by finding the extreme of the functional Equation (7) with the secondary constraint in Equation (42).

If f(x) is an odd function, then based on Equation (42):

(44) $$\iint_{-\infty }^{\infty }x_{1}^{k}x_{2}^{l}\left(-\frac{\partial (x_{2}p)}{\partial x_{1}}+\frac{\partial }{\partial x_{2}}\left(\left(2\omega_{b}x_{2}+\omega_{0}^{2}x_{1}+f\left(x_{1}\right)\right)p\right)+\frac{1}{2}K\frac{\partial^{2}p}{\partial x_{2}^{2}}\right)dx_{1}dx_{2}=0,$$

from which and after several adjustments, we obtain:

(45) $$\iint_{-\infty }^{\infty }P_{kl}\left(x_{1},\;x_{2}\right)\cdot p\left(x_{1},\;x_{2}\right)dx_{1}dv_{x}=0$$

(46) $$P_{kl}\left(x_{1},x_{2}\right)=kx_{1}^{k-1}x_{2}^{l+1}-2l\omega_{b}x_{1}^{k}x_{2}^{l}-l\omega_{0}^{2}x_{1}^{k+1}x_{2}^{l-1}-lx_{1}^{k}x_{2}^{l-1}f\left(x_{1}\right)+\frac{1}{2}sl\left(l-1\right)x_{1}^{k}x_{2}^{l-2}.$$

The whole functional with an unconstrained extreme has the form:

(47) $$S^{*}=-\iint_{-\infty }^{\infty }p\cdot \mathrm{lg}\;p\cdot dx_{1}dx_{2}-\left(\lambda_{0}-1\right)\iint_{-\infty }^{\infty }pdx_{1}dx_{2}-\iint_{-\infty }^{\infty }p\sum_{k,l=1}^{\infty }\lambda_{kl}\cdot P_{kl}\left(x_{1},x_{2}\right)dx_{1}dx_{2},$$

and thus the extreme follows as:

(48) $$p\left(x_{1},x_{2}\right)=C\cdot \exp\left(-\sum_{k,l=1}^{\infty }\lambda_{kl}\cdot P_{kl}\left(x_{1},x_{2}\right)\right)$$

and $\lambda_{0}$ is included in the normalization constant *C*. The constants $\lambda_{kl}$ can be obtained by re-substituting Equation (48) into Equation (46). After analogous operations to those in Section 4.1, a similar procedure to that described in Section 4.1 brings us from Equation (48) to Equation (43).

**Figure 8:**
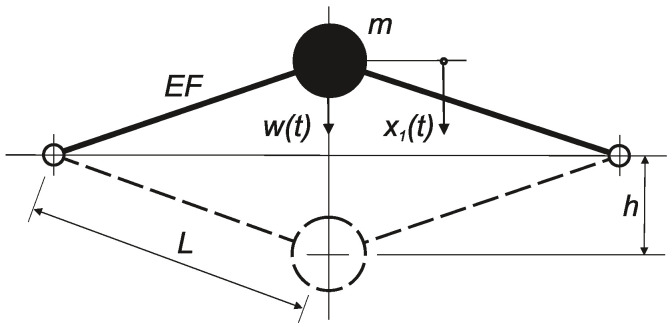
Mieses strutted frame as a nonlinear SDOF system with white noise excitation.

Next, we demonstrate this procedure based on the Mieses strutted frame with one degree of freedom, as shown in Figure 8. The fact that the system exhibits the snap-through effect does not affect the numerical aspects of the procedure in terms of the numerical stability or convergence velocity. The governing system reads:

(49) $$\begin{array}{c} \dot{x_{1}}=x_{2}\\ \dot{x_{2}}=\frac{1}{m}\left(-2bx_{2}-K\left(2h^{2}x_{1}-3hx_{1}^{2}+x_{1}^{3}\right)\right)+\frac{w(t)}{m}, \end{array}$$

where:

- *h*—Mieses strutted frame rise,
- $K=EF/L^{3}$—“longitudinal stiffness” of one bar of the strutted frame,
- *m*—concentrated mass in the movable node of the strutted frame.

If w(t) is a stationary white noise, the relevant FPE has the form:

(50) $$-\frac{\partial x_{2}p}{\partial x_{1}}+\frac{\partial }{\partial x_{2}}\left(\left(\frac{2b}{m}x_{2}+\frac{K}{m}\left(2h^{2}x_{1}-3hx_{1}^{2}+x_{1}^{3}\right)\right)p\right)+\frac{s}{2m^{2}}\frac{\partial^{2}p}{\partial x_{2}^{2}}=0.$$

Equation (50) has an analytic Boltzmann solution (e.g., as described by [12] or [23]):

(51) $$p\left(x_{1},x_{2}\right)=C\cdot \exp\left(-\frac{2bm}{s}\cdot x_{2}^{2}\right)\cdot \exp\left(-\frac{4bK}{s}\cdot x_{1}^{2}{\left(\frac{1}{2}x_{1}-h\right)}^{2}\right),$$

where *C* is a normalization constant (see Equation (48)).

This solution can be obtained based on the maximum of the only functional Equation (7) with the secondary constraints in Equation (50). If we consider q=4 terms of the infinite series in Equation (48), then for the moments, we can write: $\mu_{kl}\left(k,l=0,\ldots ,4;\;k+l\leq 4\right)$, with 62 equations in total for 15 unknown quantities. As an example, we only select the first two of these equations to obtain the values of the unknown quantities that differ from zero:

(52) $$\begin{array}{cc} \frac{2b}{m}-\frac{s}{m^{2}}\mu_{20}+\frac{s}{2m^{2}}\mu_{10}^{2} & =0\\ 2\mu_{02}-\frac{2b}{m}\mu_{11}-\frac{4Kh^{2}}{m}\mu_{20}-\frac{3s}{m^{2}}\mu_{31}+\frac{2s}{m^{2}}\mu_{10}\mu_{21}+\frac{2s}{m^{2}}\mu_{11}\mu_{20} & =0\\ \mathrm{etc}. & \end{array}$$

Equation (52) have the following solution:

(53) $$\mu_{20}=\frac{2bm}{s};\;\mu_{02}=\frac{4bKh^{2}}{s};\;\mu_{03}=-\frac{4bKh}{s};\;\mu_{04}=\frac{bK}{s},$$

which coincides perfectly with the Boltzmann solution. Other values of $\mu_{kl}$ vanish, except for $\mu_{00}$, which serves as the normalization constant, $\exp(\mu_{00})=C$, and it is also determined after Equation (48) has been cleaned of all terms where $\mu_{kl}$ is equal to zero. Equation (49) comprise a typical system with a marked non-Gaussian bi-modal response. Maximizing the functional in Equation (7) using Equation (47) with the known polynomials $P_{kl}\left(x_{1},\;x_{2}\right)$ to search for the unknown multipliers $\lambda_{kl}$ yields the final result. The solution to Equation (48) characterizes this situation well and leads to the known classical solution, which confirms the validity of the maximum entropy principle for determining the PDF of a dynamic system. The numerical experiments also confirmed the rapid convergence with q=4 as a satisfactory value to match the Boltzmann solution with Equation (51). Stochastic simulation was also used as an independent tool for verification. Careful numerical processing should be performed due to the stochastic character of the original stochastic differential system in terms of integration stepping and the corrector steps for improving the stability (e.g., see [56]).

## 6. Dynamic Systems with Many Degrees of Freedom

### 6.1. General Formulation

The motion of a system of concentrated masses with *n* degrees of freedom and potential links is described by Equation (1). In order to maximize the functional *S* according to Equation (7) and with the secondary constraints in Equation (8) in either the direct form of the FPE or the form of stochastic moments, we can investigate any admissible dynamic system response PDF. Nevertheless, as shown in the previous sections, the maximization techniques for particular cases can be complicated and ineffective, especially when n>2 and higher. However, some special classes of dynamic systems allow significant simplification despite a high value of *n*, particularly when the stochastic moments are convergent and this allows us to approximate the respective PDF with any arbitrary precision.

The original system Equation (1) can be rewritten in the form:

(54) $$\begin{array}{cc} {\dot{x}}_{1} & =x_{2}\\ m{\dot{x}}_{2} & =-2\varepsilon x_{2}-\frac{\partial U}{\partial x_{1}}+Gw \end{array}$$

where:

- $m\in R^{n\times n}$—diagonal square matrix of concentrated masses $m_{j}$ acting in individual degrees of freedom,
- $\varepsilon \in R^{n\times n}$—diagonal matrix of viscose damping coefficients $\varepsilon_{j}$,
- $x_{1},\;x_{2}\in R^{n}$—vectors of displacement or velocity, respectively, in individual degrees of freedom,
- $w\left(t\right)\in R^{m}$—vector of white noise with intensity $s_{j}$ applied as excitation forces
- $G\in R^{n\times m}$—rectangular matrix transforming white noise w into relevant degrees of freedom,
- $U=U(x_{1})$—potential energy (scalar function of displacements $x_{1}$) of the system.

For example, Equation (54) can model the motion of a system of concentrated masses with large amplitudes if the individual masses are mutually interconnected by massless springs and the damping is affected by a “slightly” viscous environment, thereby allowing the definition of a framework with hinged nodes if the masses of the bars are concentrated in nodes.

The basic idea is obvious, i.e., searching for an alternate system (AS) that is close to the original and that exhibits some special properties to allow radical simplification in terms of degrees of freedom. In particular, this means that subsystems (precisely or approximately) can occur that work more or less independently of the remaining parts of the AS to provide a suitable functional basis (forming a separable functional space in the ideal case). If any can be found, the probabilistic problem can be solved based on the AS using suitable conventional solution methods. The result, such as a one point $p_{0}\left(x\right)$ or multiple point $p_{0i}\left(x\right)$ basis, then serves as a zero approximation when using the maximum entropy probability principle in the decomposition of stochastic moments. We outline two possibilities in the next two subsections.

### 6.2. Stochastically Proportional Systems

Provided that the conditions:

(55) $$m_{j}=\mathrm{const}.;\;\varepsilon_{j}/s_{j}=\eta =\mathrm{const}.$$

are satisfied it is possible to use the methods described in the previous sections to find the maximum of the functional Equation (7) with secondary constraints constructed based on Equation (54). The result is the formula corresponding to the Boltzmann solution (see [23]):

(56) $$p\left(x_{1},x_{2}\right)=\frac{1}{J}\exp\left(-4\eta \left(U+K\right)\right),$$

where $K=K(x_{1},x_{2})$ is the kinetic energy of the system.

It should be noted that the satisfied constraints leading to the PDF according to Equation (56) indicate the stochastic independence of the velocities $x_{2}$ with respect to the displacements $x_{1}$, which confirms the validity of the Heisenberg principle of indeterminacy. A system endowed with the property in Equation (55) can be referred to as a stochastically proportional system (SPS).

A system of equations for the unknown parameters λ with a higher number of degrees of freedom can only be constructed with a computer. The solution is complicated because the system of equations is nonlinear. The system and its solution can be simplified if the system approaches the proportional Hamiltonian system. In this case, it is possible to write the Boltzmann solution and use it as the initial approximation for the gradient method of the solution to the system for the parameters λ. These ideas form the basis of a computer program that generates a nonlinear system of equations for computing the parameters λ for a given geometry of the system (coordinates of nodes, rigidities $C_{k}$, masses $m_{j}$, damping matrices ε, and incidence tables T) considered as an oriented graph, excitation intensities K (diagonal in a special case), and the upper limit *q* of the degree of the polynomial in Equation (48). At present, the program only works for stationary cases, i.e., with constant $s_{j}$. Therefore, the system of equations is algebraic and can be solved using standard methods.

The original code was assembled in order to verify the general strategies described above. The test examples are rather hypothetical. In order to fulfill the conditions in Equation (55), damping parameters $\epsilon_{j}$ and white noise intensities $s_{j}$ were selected and the AS was examined. Next, settings that differed slightly from the conditions in Equation (55) were employed and the entropy probability was maximized.

Two systems were examined as follows.

**Figure 9:**
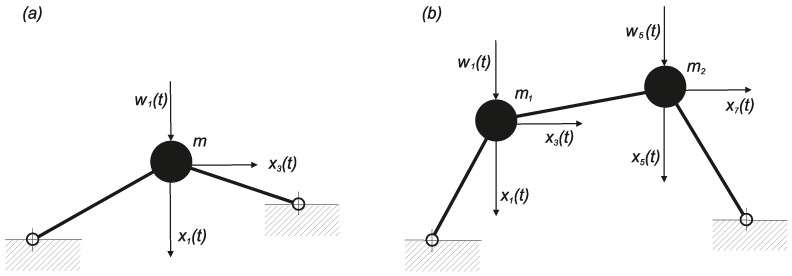
Testing strutted Mieses type frames with white noise excitation: (**a**) system with two degrees of freedom; (**b**) system with four degrees of freedom.

1. The Mieses strutted frame with one mass and two degrees of freedom, as shown in Figure 9a, while only considering the SDOF system (vertical displacement) described in Section 5.2. At the level of q=4, the algebraic system contained 70 unknown parameters λ and most of them equaled zero. Compared with Equation (55), the results did not differ qualitatively where the stochastic coherence velocity/displacement was still negligible, although the excitation acted only on one degree of freedom.
2. A non-symmetrical strutted frame with two masses and four degrees of freedom, as shown in Figure 9b, at the level of q=4. Again, the velocity and displacement interaction was negligibly small (it was impossible to determine based simply on the approximate character of the overall solution process). The stochastic relationship of the individual displacements varied considerably in terms of the dependence on the excitation intensity. This relationship was small when the excitation was generally small and local snap-through did not occur with high probability. However, it increased steeply locally or globally immediately after overcoming some energy barrier that kept the motion within local limits. The signs of the coefficients λ for the fourth powers of the polynomial in the exponent of the function $p(x_{1},\;x_{2})$ were positive. Therefore, the boundary conditions in Equation (11) were satisfied without difficulty.

### 6.3. Transformation of the System with Respect to Nonlinear Normal Modes

Another suitable strategy appears to be using a combination of the maximum entropy probability principle with the preliminary involvement of NNM transformation. NNM was first proposed by Rosenberg [57] and extended subsequently (see [58,59]).

Analogous to the classical linear eigenmodes, the NNM is a tool for expressing the dynamic response of a system in certain generalized coordinates, which are energetically separated as far as possible from each other and thus they exhibit minimal interaction. If introduced correctly, they can be investigated individually and subsequently evaluated in terms of their interaction. At present, some commercial codes for dealing with the NNM are available. However, care should be taken because NNM can be “cheated” due to multivalent meanings. Therefore, local modes should be avoided. In general, a suitable manifold should be composed. If it is constructed successfully, the NNM can provide good quality results when used as zero iteration points for probability entropy maximization. A well separated NNM can lead to the very rapid convergence of the stochastic moments to obtain the final PDF of the system response. This is beneficial and the NNM strategy is actually used in earthquake engineering, and thus subsequent stochastic analysis based on entropy probability maximization can be facilitated by the NNM based on the results of previous activities.

## 7. Conclusions

Every earthquake event is a random process with a specific character. The occurrence of whatever seismic shock and a relevant ground motion at a particular site is not predictable. Therefore, the seismic process should be unavoidably considered as a stochastic process. This attribute has been adopted in the earthquake engineering many years ago and, therefore, we have a rich material providing wide possibilities to evaluate adequate dynamic characteristics of a stochastic nature.

In order to employ effectively collected data advanced methods of stochastic analysis of dynamic systems must be available. The method based on the maximum entropy probability density principle is worth to be considered. It seems to be very flexible from the viewpoint of structural variability and, moreover, it makes possible to intervene into parametric areas which are inaccessible for other methods. Therefore, this method enables also to serve as a tool for verification of results earned using other methods. It concerns predominantly structures which can be modeled as MDOF systems with concentrated masses, as it is a typical case of structures made of 1D elements (frameworks, trusses, etc.). Furthermore, all systems with well pronounced cyclic structure, easily transformable into state with well separable NNM, or systems close to those enabling closed form solutions of Boltzmann type are appropriate to be analyzed using this way. Many structures of this type can be encountered in industry and traffic engineering.

The problem of finding the probability density for a random movement of a system due to external random excitation can be considered as the problem of finding the constrained maximum of the functional of the entropy of the probability density. In the case of a system with polynomial nonlinearities, these properties of the extreme can satisfy the secondary constraints derived from the FPE and they may be described best by a multidimensional exponential function with the exponent in the form of a complete polynomial of a certain degree with unknown coefficients. This can be determined from the FPE equation for the system considered by using the stochastic moment decomposition strategy. Our comparison of the results with analytically solvable cases demonstrated their good agreement, with very rapid convergence of the first stochastic moments for the PDF approximation. The advantage of this procedure is the certainty of the positive probability density values in the whole phase space. The disadvantage is the great number of unknown parameters λ, and the need to monitor their positive sign in the highest powers of the phase variables. In the opposite case, the problem loses stochastic stability in terms of the probability. The test cases based on simple MDOF systems indicated good numerical feasibility, although the test cases were located in close proximity to proportional systems or in the neighborhood of the dominant NNM.

Our general analysis and the results obtained for some particularly simple cases demonstrated how the response of a nonlinear system differed significantly from a Gaussian process even though the excitation had Gaussian characteristics. In addition, the principle considered in this investigation allowed us to prove that according to the number of processes with equal dispersal, a very normal process has the probability density with the highest entropy. Thus, the system always tends to produce a Gaussian response if it is permitted by the internal structure of the system. This is also the reason why Gaussian processes occur most frequently in nature.

An open problem comprises the possible existence of a greater number of extremes for a single fixed input and the practical procedure for finding them, particularly with respect to their initial approximation. This problem requires the prior elimination of some parameters λ because their number increases exponentially as both the number of degrees of freedom and the level of *q* increase. Experience indicates that most of the parameters λ have values that are either equal to zero or insignificant. Thus, the related stochastic moments are either negligible or they vanish. In addition, the problem of how to select the equations remains unsolved, where the number for a given level of *q* is several times higher than the number of unknown parameters λ. Some of these problems are closely related and they should be solved as a single group.

Obvious difficulties occur due to the increase in the number of independent phase variables $x_{j}$ as the degrees of freedom increase in the system considered. However, these problems affect all methods for solving the FPE unless they are developed for a narrow class of systems with special properties, or if searching for only some special attributes of the solution. In the maximum entropy probability method, these cases can be considered as systems with well separated generalized coordinates by using the NNM in the preliminary step, or systems that are close to those that can be solved exactly, e.g., by employing the Boltzmann solution or systems with very weak nonlinearity. When searching for suitable classes of systems, this step is related to each specific FPE solution method or the qualitative investigation. An important strength of the strategy based on maximizing the probability entropy is the fact that it affects different classes where it has been proved effective, and thus it complements a set of existing methods for solving or investigating the FPE.

## Abbreviations

The following abbreviations are used in this manuscript:

FEM	Finite Element Method
FPE	Fokker–Planck Equation
MDOF	Multi-Degree of Freedom
SDOF	Single Degree of Freedom
PDF	Probability Density Function
NNM	Nonlinear Normal Mode
SPS	Stochastically Proportional System
AS	Alternate System
